# The Destruction of Medical Certificates

**Published:** 1917-09-29

**Authors:** 


### The Destruction of Medical Certificates.

Not infrequently newspapers report complaints made to the military tribunals that medical certificates or other documents—papers often of great value to men of military age—have gone astray while in the hands of the military authorities to whom they have been submitted. In the pressure of work which necessarily occurs in connection with the raising and classifying of a huge army mistakes will occur, but the case of *Charlesworth* v. *Lieut.-Colonel Fryer* was entirely outside of that excuse, and is worthy of record. The plaintiff was an applicant for exemption under the Military Service Acts. He had been examined by a medical board and classified C.1. He showed to the board three certificates by local practitioners of repute, and on the conclusion of his examination asked for the return of the certificates. A civilian doctor member of the board handed them to him, whereupon the defendant, an R.A.M.C. officer, president of the board, took them from him and tore them up, remarking that they might "be used by someone else." The plaintiff sued the defendant for unlawful trespass to personal property, and averred that defendant's action was illegal and a violation of the rights of the appellant, who, in fact, required the certificates for his appeal to the tribunal; and further that the insinuation that the plaintiff might fraudulently dispose of the certificates was in effect libellous. The defendant, who admitted the facts, stated that it was his practice to tear up medical certificates to prevent fraud; but the court found the defendant liable.

